# Oridonin, a novel lysine acetyltransferases inhibitor, inhibits proliferation and induces apoptosis in gastric cancer cells through p53- and caspase-3-mediated mechanisms

**DOI:** 10.18632/oncotarget.8033

**Published:** 2016-03-10

**Authors:** Min Shi, Xiao-Jie Lu, Juan Zhang, Hua Diao, Guangming Li, Ling Xu, Ting Wang, Jue Wei, Wenying Meng, Jia-Li Ma, Heguo Yu, Yu-Gang Wang

**Affiliations:** ^1^ Department of Gastroenterology, Shanghai Tongren Hospital, Affiliated to Shanghai Jiao Tong University School of Medicine, Shanghai, China; ^2^ Department of Radiology, Zhong-da Hospital, Medical School, Southeast University, Nanjing, China; ^3^ Department of Rehabilitation, The Affiliated Huai'an Hospital of Xuzhou Medical College and The Second People's Hospital of Huai'an, Huai'an, China; ^4^ NPFPC Key Laboratory of Contraceptives and Devices, Shanghai Institute of Planned Parenthood Research (SIPPR), Institutes of Reproduction and Development, Fudan University, Shanghai, China; ^5^ Department of Gastroenterology, Xinhua Hospital, Shanghai Second Medical University, Shanghai, China

**Keywords:** oridonin, lysine acetyltransferase inhibitor, gastric cancer, antiproliferation, apoptosis

## Abstract

Lysine acetylation has been reported to involve in the pathogenesis of multiple diseases including cancer. In our screening study to identify natural compounds with lysine acetyltransferase inhibitor (KATi) activity, oridonin was found to possess acetyltransferase-inhibitory effects on multiple acetyltransferases including P300, GCN5, Tip60, and pCAF. In gastric cancer cells, oridonin treatment inhibited cell proliferation in a concentration-dependent manner and down-regulated the expression of p53 downstream genes, whereas p53 inhibition by PFT-α reversed the antiproliferative effects of oridonin. Moreover, oridonin treatment induced cell apoptosis, increased the levels of activated caspase-3 and caspase-9, and decreased the mitochondrial membrane potential in gastric cancer cells in a concentration-dependent manner. Caspase-3 inhibition by Ac-DEVD-CHO reversed the proapoptosis effect of oridonin. In conclusion, our study identified oridonin as a novel KATi and demonstrated its tumor suppressive effects in gastric cancer cells at least partially through p53-and caspase-3-mediated mechanisms.

## INTRODUCTION

Gastric cancer is one of the most common cancers and the second leading cause of cancer-related deaths worldwide [1]. Up to now, there remains a lack of satisfactory therapeutic agents for patients with advanced gastric cancer [2-3] and thus the development of novel and effective therapeutic agents is still urgently needed.

Oridonin is a natural diterpenoid isolated from the Chinese medicinal herb *Rabdosia rubescens* (or *Donglingcao* in Chinese), which has been used to treat patients with gastric cancer for many years in traditional Chinese medicine [4, 5]. It has been reported to have tolerable toxicities *in vivo* [6] and possess anti-tumor activities in a variety of human malignancies such as leukemia [7], liver cancer [8], pancreatic cancer [9], fibrosarcoma [10] and cervical cancer [11]. In our preliminary study, oridonin was found to possess acetyltransferase-inhibitory effects. Protein acetylation and its reverse process called deacetylation are dependent on the activities of two key enzymes termed lysine acetyltransferases (KATs) and lysine deacetylases (KDACs) [13, 14]. Abnormal lysine acetylation induced by the imbalance of the two enzymes has been reported to involve in the pathogenesis of multiple diseases including cancer [12-15]. During the past decades many clinical trials on KDAC inhibitors (KDACi) have been conducted worldwide [17] and mechanisms underlying the tumor suppressive effects of KDACi have been studied extensively [18, 19]. On the contrary, only few KATis have been identified. Although some KATis have showed oncosuppressive effects, such as anacardic acid [20], epigallocatechin-3-gallate (EGCG) [21], curcumin [22] and garcinol [23], none of them possess the required efficacy, specificity and tolerability to be developed as anticancer agents [24].

In this study, we identified oridonin as a novel and effective KATi and investigated its effects on the proliferation and apoptosis of human gastric cancer cells. We also set foot in the exploration of the underlying mechanisms to provide impetus for future studies.

## RESULTS

### Inhibitory effects and preferred targets of oridonin on acetyltransferase activity

**Figure 1:**
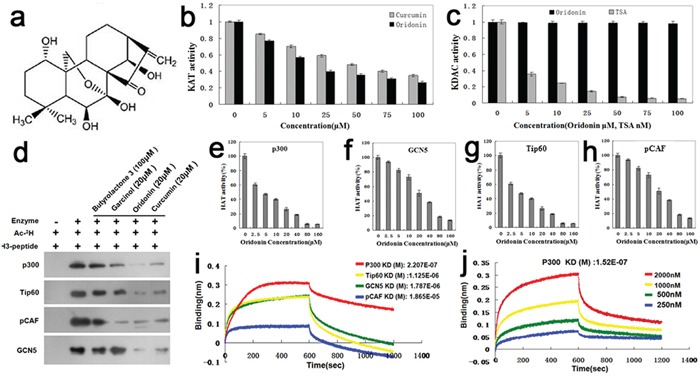
Identification of oridonin as a novel acetyltransferase inhibitor **a.** Structure of oridonin: oridonin belongs to the ene-kaurane diterpenoids. **b.** Comparison of the KATi activity of oridonin with that of a known KAT inhibitor curcumin by using the KAT Activity Colorimetric Assay Kit. Bars represent means±S.E.M. (n=3). **c.** Comparison of the KDACi activity of oridonin with that of a known KDAC inhibitor TSA by using the KDAC Inhibitor Drug Screening Kit (Fluorometric) (BioVision). Bars represent means±S.E.M. (n=3). **d.** The *in vitro* inhibitory effect of oridonin on acetylation of histone 3 was validated by autoradiography. The KATi effects of Oridonin on p300, Tip60, Pcaf and GCN5 were compared with those of established KATis such asbutyrolactone 3, curcumin and garcinol. (e-h) Assays of the KATi activities of Oridonin on P300 **e.** GCN5 **f.** Tip60 **g.** and pCAF **h.** Bars represent means±S.E.M. (n=4). **i.** The equilibrium dissociation constants between oridonin and the four acetyltransferases (P300, GCN5, Tip60 and pCAF) were measured by the ForteBio Octet RED96 system. **j.** The equilibrium dissociation constant kD (M) between oridonin and P300 of different concentrations (250, 500, 1000 and 2000 nM).

Oridonin (Figure 1a) is a member of the ene-kaurane diterpenoids. Oridonin inhibited KAT activity (Figure 1b) but had no effects on the activity of KDACs (Figure 1c). As shown in Figure 1b, the inhibitory effects of oridonin on KAT activity were more potent than those of curcumin (an established KATi) at the same concentrations (p<0.05). Oridonin can inhibit multiple acetyltransferases including P300, GCN5, Tip60 (Tat-interacting protein 60) and PCAF (P300/CBP-associated factor) in a dose-dependent manner (Figures 1d–1h). It was particularly potent in inhibiting P300, with an IC_50_ of ∼5 μM (Figure 1e). Moreover, the KATi activity of oridonin was more potent than those of established KATis such as butyrolactone 3, curcumin and garcinol (Figure 1d).

As reflected by equilibrium dissociation constants that were measured with the ForteBio Octet RED96 system, the binding affinities of oridonin to the four acetyltransferases tapered off as following: P300 > Tip60 > GCN5 > pCAF (Figure 1i). Figure 1j shows the overall equilibrium dissociation constant KD (M) between oridonin and P300 of different concentrations (250, 500, 1000 and 2000 nM).

### Effects of oridonin on proliferation and apoptosis of gastric cancer cells

**Figure 2:**
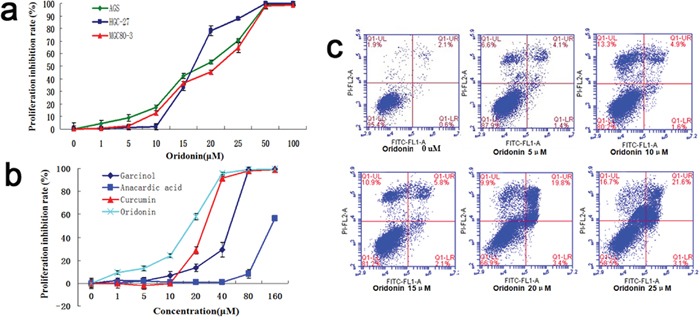
Effects of oridonin on theproliferation and apoptosis of gastric cancer cells **a.** Inhibitory effects of oridonin on the proliferation rate of human gastric cancer cell lines (AGS/HGC-27/MGC80-3) in a concentration-dependent manner. Bars represent means±S.E.M., n=3. **b.** Oridonin is more potent in inhibiting cell proliferation than other KATis including butyrolactone 3, curcumin and garcinol. Bars represent means±S.E.M., n=3. **c.** Apoptosis assays of AGS cells by flow cytometry upon oridonin treatment (concentration gradient: 0, 5, 10, 15, 20 and 25 μM) showed that oridonin induced cell apoptosis in a concentration-dependent manner.

After treatment with oridonin of different concentrations (0, 1, 5, 10, 15, 20, 25, 50 or 100 μM) for 48 h, the proliferation rates of three human gastric cancer cell lines (AGS, HGC-27 and MGC80-3) were assessed by the CCK-8 kit. As shown in Figure 2a, oridonin possessed anti-proliferative effects on all the three cell lines in a concentration-dependent manner, with AGS being the most sensitive one to oridonin of low concentrations (5–15 μM). Therefore, AGS cell was chosen for subsequent experiments.

Next we compared the anti-proliferative effects on AGS cells of oridonin and those of three other KATis (butyrolactone 3, curcumin and garcinol). As shown in Figure 2b, all of the four KATis exhibited anti-proliferative effects whereas oridonin was the most potent one, especially at low concentrations (10–20 μM).

To investigate the effects of oridonin on AGS cell apoptosis, we subjected oridonin-treated AGS cells to flow cytometry analysis, the results of which showed that the proportion of apoptotic cells increased upon oridonin treatment in a concentration-dependent manner (0, 5, 10, 15, 20 and 25 μM), indicating that oridonin induces apoptosis in AGS cells (Figure 2c).

### Effects of oridonin on the gene expression profiles of AGS cells

**Figure 3:**
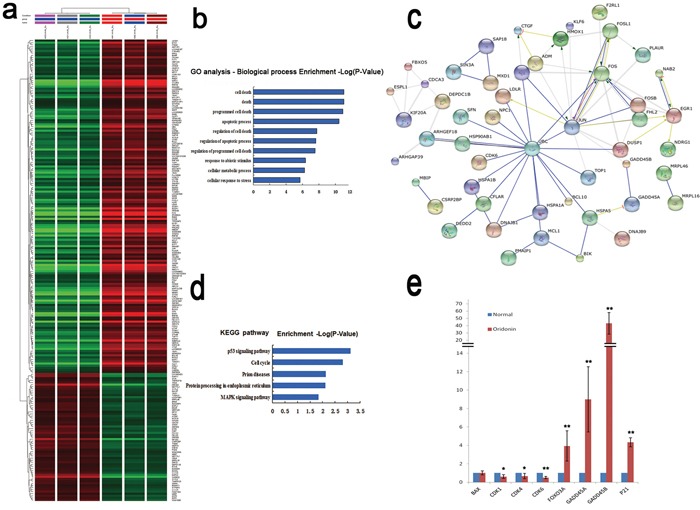
Effects of oridonin on the gene expression profiles in AGS cells assessed by microarray **a.** Clustering analysis of the DEGs upon oridonin treatment using Gene Spring software. Gene expression profiles of AGS cells with or without oridonin treatment were assayed by microarray. **b.** GO analysis of DEGs for biological processes. **c.** Interactions among the DEGs revealed by KEGG pathway analysis. **d.** DEGs enriched in KEGG pathways. **e.** The relative mRNA expression levels of some p53 downstream genes in AGS cells with or without oridonin treatment revealed by microarray. Bars represent means±S.E.M. (n=3). DEGs: Differently expressed genes; GO: Gene Ontology; KEGG: Kyoto encyclopedia of Genes and Genomes.

The total mRNAs of AGS cells treated with oridonin (15μM) or vehicle alone were isolated and analyzed using gene expression microarray to profile their global gene expression patterns. Through cluster analysis and interaction network analysis, 221 differentially expressed genes (DEGs) were identified in oridonin-treated cells versus control cells. Of the 192 genes with fold changed ≥2, 141 were up-regulated whereas the other 51 down-regulated (Figure 3a). Gene Ontology (GO) analysis revealed that these DEGs were highly enriched in cell death and apoptosis-related biological processes (Figure 3b). Kyoto encyclopedia of Genes and Genomes (KEGG) pathway analysis showed the enrichment of DEGs in several pathways including the p53 signaling pathway and the mitogen-activated protein kinase (MAPK) signaling pathway (Figure 3c, 3d). Moreover, the expression of several downstream genes of p53 changed upon oridonin treatment, with CDK1, CDK4 and CDK6 downregulated and FOXO3A, GADD45A, GADD45B and p21 upregulated (Figure 3e).

### Involvement of the p53 signaling pathways in the anti-proliferative effect of oridonin on AGS cells

**Figure 4:**
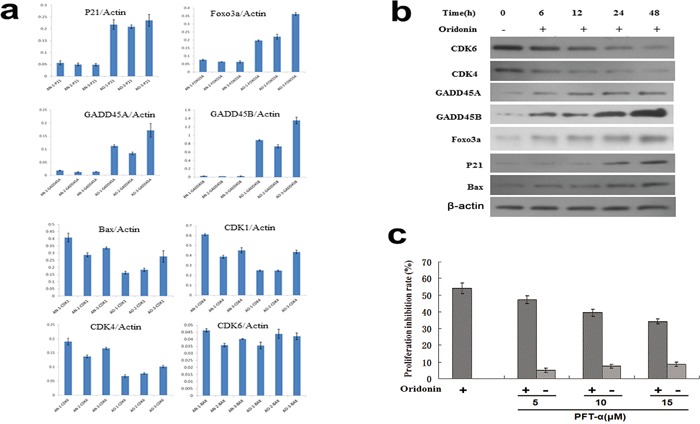
Validation of representative DEGs identified by gene microarray analyses by qPCR and western blot **a.** qPCR assays of the expression levels (normalized to actin) of p21, FOXO3A, GADD45A, GADD45B, Bax CDK1, CDK4 and CDK6. Bars represent means±S.E.M. (n=3). **b.** Western blot showed that the expression levels of FOXO3A, GADD45A, GADD45B, p21 and Bax increased whereas those of CDK1, CDK4 and CDK6 decreased upon oridonin treatments for 48 hours in AGS cells. **c.** p53 inhibitor PFT-α partially conteracts the anti-proliferative effect of oridonin on AGS cells in a concentration-dependent manner. Bars represent means±S.E.M. (n=4). *:P<0.05; **P<0.01; DEGs: differently expressed genes; qPCR: quantitative polymerase chain reaction.

To validate the results of gene microarray, expressions of DEGs in the p53 signaling pathway were assayed by quantitative reverse-transcription polymerase chain reaction (RT-qPCR) (Figure 4a) and western blots (Figure 4b) in oridonin-treated AGS cells and controls. As shown in Figure 4, the expression levels of CDK1, CDK4 and CDK6 decreased and the expression levels of FOXO3A, GADD45A, GADD45B and p21 increased upon oridonin treatment. These results were in line with the results of microarray analysis. The protein expression levels of CDK4, CDK6, FOXO3A, GADD45A, GADD45B, and p21 were consistent with results of qPCR. Interestingly, the protein level of Bax increased in the oridonin-treated group whereas its mRNA showed no significant change upon oridonin treatment (Figure 4a, 4b).

**Figure 5:**
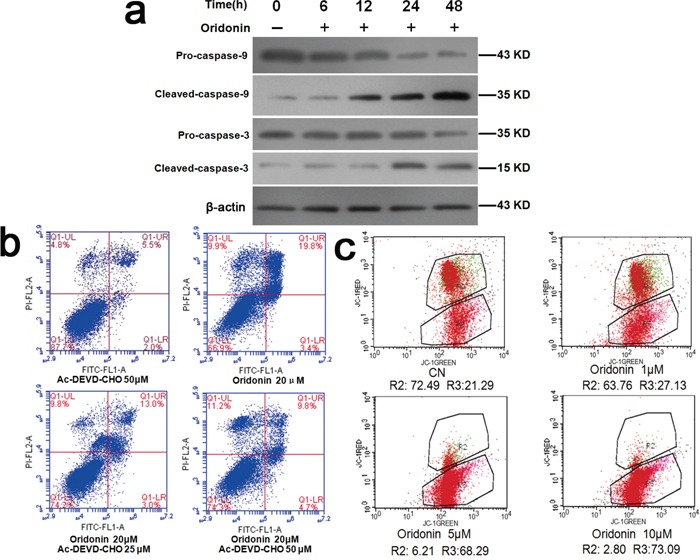
Explorations into the mechanisms underlying the proapoptosis effects of oridonin **a.** Western blots showed that oridonin treatment increased the expression levels of activated caspase-3 and activated caspase-9 whereas had no significant effects on those of caspase-3, caspase-9 in AGS cells. **b.** Flow cytometry analyses of the apoptosis-inducing effect of oridonin on AGS cells with and without caspase-3 inhibitor Ac-DEVD-CHO. Oridonin treatment induced apoptosis, which is partially counteracted by Ac-DEVD-CHO. **c.** Flow cytometry analyses of the mitochondrial membrane potential of AGS cells treated with oridonin of different concentrations. AGS cells were stained with JC-1. R2: District for living cells; R3: District for apoptotic cells. CN: control; JC-1: a kind of fluorescent probe for detecting mitochondrial membrane potential. Its molecular formula: C25H27Cl4IN4.

Next, a p53 inhibitor called PFT-α was used to block p53 function. As shown in Figure 5b, the anti-proliferative effect of oridonin on AGS cells was attenuated upon PFT-α treatment in a concentration dependent manner, confirming that the anti-proliferative effect of oridonin is at least partially mediated by p53-dependent mechanisms (Figure 4c).

### Involvement of caspase-3 and mitochondrial membrane potential in the proapoptosis effect of oridonin in AGS cells

As shown in Figure 5a, oridonin elevated the levels of activated caspase-3 and activated caspase-9 in AGS cells in a concentration-dependent manner (p<0.05) while sparing the precursors of these two caspases. Flow cytometry revealed that the proapoptosis effect of oridonin on AGS cells was attenuated by a caspase-3 inhibitor called Ac-DEVD-CHO, suggesting that oridonin induces apoptosis at least partially through caspase-3-mediated pathway (Figure 5b).

Decline in mitochondrial transmembrane potential is implicated in the activation of apoptosis [25, 26]. Flow cytometry of oridonin-treated AGS cells revealed that oridonin lowered the mitochondrial membrane potential of AGS cells in a concentration-dependent manner (0, 1, 5 and 10 μM) (p<0.01) (Figure 5c), suggesting that reducing mitochondrial transmembrane potential might be another mechanism of the pro-apoptosis effect of oridonin.

## DISCUSSION

In this study, we found that oridonin is a novel KATi. It can inhibit multiple acetyltransferases and exhibited oncosuppressive effects on gastric cancer cells. We found that oridonin is more potent in inhibiting KAT activity and the proliferation of gastric cancer cells than previously reported KATi such as butyrolactone 3, curcumin and garcinol (Figure 1d, 2b). These results identified oridonin as a useful agent for the functional investigation of cellular acetylation events and suggested the potential of oridonin to be developed as a preventive or therapeutic agent for gastric cancer in the future.

Besides revealing the oncosuppressive effects of oridonin, our study also explored the possible mechanisms underlying these effects. Global gene expression and pathway analysis showed that the DEGs upon oridonin treatment were enriched in the p53 signaling pathway (Figure 3d). Oridonin treatment changed the expression levels of multiple downstream genes of p53 (Figure 3e, 4a, 4b) and blockage of p53 function by its inhibitor PFT-α attenuated the antiproliferative effects of oridonin in a concentration-dependent manner (Figure 4c). All these results pointed to the conclusion that the antiproliferative effect of oridonin is at least partially p53-dependent. Previous studies have suggested that acetylation and deacetylation of p53 are involved in different biological processes at transcriptional level [27, 28]. For example, acetylation of p53 by P300/CBP can activate the sequence-specific DNA-binding activity of p53, which mediates its antiproliferative effects [29]. We found that upon oridonin treatment, p53 K382 acetylation level was downregulated whereas p53 S392 phosphorylation level was upregulated in AGS cells (data not shown), but it is still an open question whether these two posttranslational events are cross-linked and whether these modifications of p53 are responsible for the p53-mediated antiprofirative effect upon oridonin treatment.

As for the mechanisms underlying oridonin-mediated proapopotosis effects, we demonstrated that oridonin treatment resulted in elevated levels of caspase-3 and caspase-9 in their activated forms (Figure 5a) and that this proapoptosis effect was attenuated by a caspase-3 inhibitor (Figure 5b), suggesting that oridonin induces apoptosis at least partially through caspase-3-mediated pathway. Mitochondrial pathway is another established regulator of apoptosis [25, 26]. Decline in mitochondrial transmembrane potential is implicated in the induction and activation of cell apoptosis [25, 26]. Our results demonstrated that oridonin treatment decreased the mitochondrial transmembrane potential (ΔΨm) (Figure 5c), which might be one of the mechanisms underlying oridonin-mediated proapoptosis effects. Previous studies have suggested that mitochondrial stress and mitochondrial biogenesis are associated with cell cycle [30, 31]. Our study revealed that oridonin can inhibit cell cycle progression and induce mitochondrial stress. An open question to be investigated in future studies is that whether these two events are directly associated or isolated.

However, our study also suffers from some limitations that need to be addressed by future studies. First, it remains unclear the roles of posttranslational modifications of p53 by oridonin in p53-mediated antiproliferative effect upon oridonin treatment. Second, the tumor suppressive effects of oridonin remain to be validated *in vivo*. A previous study [32] has shown that oridonin can suppress tumor formation in xenograft model of gastric cancer cells when delivered via intraperitoneal injection. However, it remains unclear whether oral administration of oridonin can result in adequate level of oridonin in the circulation to be effective in the treating gastric cancer.

In conclusion, our study for the first time identified oridonin as a novel acetyltransferase inhibitor and demonstrated that oridonin can inhibit proliferation and induce apoptosis in AGS cells in a concentration-dependent manner. We showed that the antiproliferative effect of oridonin is at least partially dependent on the p53 signaling pathway and that oridonin can modify the acetylation status of p53. Moreover, our results demonstrated that oridonin induces apoptosis at least partially through caspase-3-mediated pathway and that oridonin can reduce the mitochondrial transmembrane potential of gastric cancer cells, which might also account for its proapoptosis effect. Our study provided the first evidence that oridonin holds the potential of being developed as anticancer drugs and may stimulate future studies in this area.

## MATERIALS AND METHODS

### Assays of inhibitory activities of oridonin on acetyltransferase and deacetyltransferase

Nuclear components were extracted from 2×10^8^ AGS cells (Cell Resource Center, Shanghai Institutes for Biological Sciences, Chinese Academy of Sciences, Shanghai, China), and the protein concentrations were quantified and titered to 2 mg/ml using the bicinchoninic acid (BCA) method. Nuclear extracts (20 μl) from AGS cells were mixed with the aqueous solutions of curcumin or oridonin (20 μl, at 5, 10, 25, 50, 75 and 100 μM). 40 μl water was added to three wells each as blank controls. The activity of lysine acetyltransferase in each well was detected with the KAT Activity Colorimetric Assay Kit (BioVision, Mountain View, CA, USA).

For the assays of inhibitory activity on deacetyltransferase, oridonin (50 μl, at 0, 5, 10, 25, 50, 75 and 100 μM) and thiol-specific antioxidant (TSA) (50 μl, at 0, 5, 10, 25, 50, 75 and 100 μM) were separately mixed at each concentration in the KDAC Inhibitor Drug Screening Kit (Fluorometric, BioVision), and the inhibitory activity of oridonin was assayed with procedures as described in the kit on the following deacetyltransferases: P300 (Fluorogenic P300 Assay Kit; BPS Bioscience, San Diego, CA, USA), GCN5 (Fluorogenic GCN5 Assay Kit; BPS Bioscience), Tat-interacting protein (Tip)60 [TIP60 (human) highly active protein; AdipoGen, Seoul, Korea], and p300/CREB-binding protein-associated factor (pCAF) (pCAF Inhibitor Screening Kit, Fluorometric; BioVision). The inhibitory effects of oridonin at final concentrations of 0, 2.5, 5, 10, 20, 40, 80 and 160 μM on each acetyltransferase were assayed, and the inhibitory activities of other KATi including butyrolactone 3, curcumin and garcinol on these acetyltransferases (P300, GCN5, Tip60 and pCAF) were also assayed.

### Validation of the KATi activity of oridonin by autoradiography

The *in vitro* inhibitory effect of oridonin on the acetylation of histone 3 was validated by autoradiography. 5 μl 5× KAT Assay Buffer [250 mM HEPES (pH 7.8) and 150 mM KCl, 1 mM EDTA, 25 mM MgCl_2_, 25 mM sodium butyrate, 10 mM DTT] were added to a microcentrifuge tube. Two microliters (2 μg/μl) of Core Histone 3 peptide, 5 μl of the diluted [3H]-acetyl CoA (0.25 mCi/ml, 0.39 mCi/μM; Amersham, Bucks, UK), 1 μl of KATi (oridonin, curcumin, garcinol 20 μM, butyrolactone 100 μM) and 5 μl KAT (100–250 ng) were added into the microcentrifuge tube. Sterile distilled water was added to a final volume of 25 μl. The contents in the tube were mixed and all the components in the bottom of the tube were collected using a microcentrifuge pulse. Reactions were incubated at 30°C for 30 min. For each assay, reaction products were resolved by 15% SDS-PAGE and analyzed by autoradiography.

### Measurement of the affinity between oridonin and the four acetyltransferases

The affinity between oridonin and acetyltransferases was measured by Fortebio's Octet RED 96 (ForteBio Inc., CA, USA) as described previously [33]. The biotin-labeling of oridonin was based on the method described by Dal Piaz et al [34]. The biotin-conjugated oridonin was diluted to 100 μg/mL in dialysis buffer (20 mM sodium phosphate, pH 7.4), and 100 μg/mL biotin served as control. The sensors (Super Streptavidin) were pre-wet in dialysis buffer for 15 min prior to use and then were loaded with biotinylated oridonin for 15 min. The measurements were carried out automatically at room temperature.

### Cell proliferation assays

Cell proliferation was assayed with the Cell Counting Kit-8 (CCK-8, Dojindo Laboratories, Kumamoto, Japan). Three human gastric cancer cell lines AGS, HGC-27 and MGC80-3 (Cell Resource Center, Shanghai Institutes for Biological Sciences, Chinese Academy of Sciences, Shanghai, China) in 27 wells were divided into nine groups of three. The culture medium in each well was replaced by complete medium containing oridonin at a final concentration of 0, 1, 5, 10, 15, 20, 25, 50 and 100 μM, respectively. 10 μl CCK-8 solution was added to each well (100 μl). For the AGS cells, we added 20 μm oridonin using the same method, together with 0, 5, 10 or 15 μM Pifithrin-α (Sigma, St. Louis, MO, USA). In addition, The AGS cells were divided into eight groups of three. The culture medium in each well was replaced by complete medium containing oridonin at a final concentration of 0, 1, 5, 10, 20, 40, 80 and 160 μM, respectively (the same for other KATis such as curcumin, anacardic acid, garcinol). After the plate was incubated for 48 h at 37°C and 5% CO2, the CCK-8 assay was performed.

### Determination of apoptosis, cell cycle, and mitochondrial membrane potential by flow cytometry

Flow cytometry (FACS Calibur and LSR™ II Flow Cytometer; BD Pharmingen) was used to determine cell cycle as described previously [35]. The results were analyzed using the cell cycle fitting software FlowJo version 6.3 (TreeStar, San Carlos, CA, USA). Briefly, 50 μM of Ac-DEVD-CHO (N-Acetyl-Asp-Glu-Val-Asp-Ala) (Sigma), 20 μM oridonin, 20 μM oridonin plus 25 μM Ac-DEVD-CHO, and 20 μM oridonin plus 50 μM Ac-DEVD-CHO were added into the four culture dishes, respectively. The dishes were then placed in a 5% CO_2_/37°C incubator for 24 h. Cells were then collected and subjected to flow cytometry to detect apoptosis. Using the above methods for AGS cells, different concentrations (0, 1, 5 or 10 μM) of oridonin were added into four culture dishes containing AGS cells. The dishes were placed in a 5% CO_2_/37°C incubator for 8 h, after which, the cells were collected and subjected to flow cytometry to detect mitochondrial membrane potential. Flow cytometry with 488-nm laser excitation was used. The green fluorescein was detected using a 525-nm longpass filter, and the red fluorescein was detected using a filter >575 nm.

### Microarray analyses of gene expression profiles of AGS cells upon oridonin treatment and validation of the DEGs by quantitative polymerase chain reaction (qPCR)

Microarray analyses of gene expression profiles of AGS cells with or without oridonin treatment were performed as previously reported [36]. For qPCR validation, total RNA were extracted from AGS cells with or without oridonin treatment (15 μM, 24 hours) treatment. Reverse transcription of the total RNA were performed according to the manufacturer's instructions (RNeasy Mini Kit; Qiagen, Valencia, CA, USA). Fluorescent qPCR was performed for P21, GADD45A, GADD45B, Bax, cyclin-dependent kinase (CDK)4, CDK6, and forkhead box (FOX)O3A for data collection and analysis.

### Western blotting

One dish of AGS cells that had been cultured for 24 h was used as the pre-dose (0 h) sample. Complete medium containing 15 μm oridonin was added to four dishes for further culture. Cells in each dish were incubated at 37°C in 5% CO_2_ for 12 and 24 h and then trypsinized to collect cells. The cells were washed twice with PBS, and the culture supernatant was removed after centrifugation. Cell lysate was added to the collected cells, which were then incubated on ice. Using the BCA protein quantitation kit, we performed SDS-PAGE, electrophoretic transfer, immune response, color development, and gel image analysis for the following proteins: GADD45B, CDK6, FOXO3A, pro-caspase-9, pro-caspase-3, cleaved caspase-3 (Abcam, Cambridge, MA, USA), cleaved caspase-9, p21, CDK4, Bax, GADD45A, and β-Actin (Sigma). Again, one dish of cells were trypsinized and then collected as the pre-dose (0 h) sample. Complete medium containing 15 μm oridonin was added to six dishes for further culture. Two dishes were treated with 10 μM U0126 and 10 μM PD98059 (Selleck Chemicals, Houston, TX, USA), respectively, and then incubated at 37°C in 5% CO_2_ for 6, 12, 18 and 24 h. The cells were trypsinized and then collected. The cells were washed twice with PBS, and the culture supernatant was removed after centrifugation. The collected cells were incubated on ice. SDS-PAGE, electrophoretic transfer, immune response, color development and gel image analysis were performed for p53, p53 (phospho S392), p53 (acetyl K382) and p53 (acetyl K120) (Abcam).
